# Gene-Based Tests of Association

**DOI:** 10.1371/journal.pgen.1002177

**Published:** 2011-07-28

**Authors:** Hailiang Huang, Pritam Chanda, Alvaro Alonso, Joel S. Bader, Dan E. Arking

**Affiliations:** 1 Department of Biomedical Engineering, Johns Hopkins University, Baltimore, Maryland, United States of America; 2 High Throughput Biology Center, Johns Hopkins University School of Medicine, Baltimore, Maryland, United States of America; 3 Division of Epidemiology and Community Health, School of Public Health, University of Minnesota, Minneapolis, Minnesota, United States of America; 4 McKusick-Nathans Institute of Genetic Medicine, Johns Hopkins University School of Medicine, Baltimore, Maryland, United States of America; University of Oxford, United Kingdom

## Abstract

Genome-wide association studies (GWAS) are now used routinely to identify SNPs associated with complex human phenotypes. In several cases, multiple variants within a gene contribute independently to disease risk. Here we introduce a novel Gene-Wide Significance (GWiS) test that uses greedy Bayesian model selection to identify the independent effects within a gene, which are combined to generate a stronger statistical signal. Permutation tests provide p-values that correct for the number of independent tests genome-wide and within each genetic locus. When applied to a dataset comprising 2.5 million SNPs in up to 8,000 individuals measured for various electrocardiography (ECG) parameters, this method identifies more validated associations than conventional GWAS approaches. The method also provides, for the first time, systematic assessments of the number of independent effects within a gene and the fraction of disease-associated genes housing multiple independent effects, observed at 35%–50% of loci in our study. This method can be generalized to other study designs, retains power for low-frequency alleles, and provides gene-based p-values that are directly compatible for pathway-based meta-analysis.

## Author Summary

Genome-wide association studies (GWAS) have successfully identified genetic variants associated with complex human phenotypes. Despite a proliferation of analysis methods, most studies rely on simple, robust SNP–by–SNP univariate tests with ever-larger population sizes. Here we introduce a new test motivated by the biological hypothesis that a single gene may contain multiple variants that contribute independently to a trait. Applied to simulated phenotypes with real genotypes, our new method, Gene-Wide Significance (GWiS), has better power to identify true associations than traditional univariate methods, previous Bayesian methods, popular L1 regularized (LASSO) multivariate regression, and other approaches. GWiS retains power for low-frequency alleles that are increasingly important for personal genetics, and it is the only method tested that accurately estimates the number of independent effects within a gene. When applied to human data for multiple ECG traits, GWiS identifies more genome-wide significant loci (verified by meta-analyses of much larger populations) than any other method. We estimate that 35%–50% of ECG trait loci are likely to have multiple independent effects, suggesting that our method will reveal previously unidentified associations when applied to existing data and will improve power for future association studies.

## Introduction

Traditional single-SNP GWAS methods have been remarkably successful in identifying genetic associations, including those for various ECG parameters in recent studies of PR interval (the beginning of the P wave to the beginning of the QRS interval) [1], QRS interval (depolarization of both ventricles) [2] and QT interval (the start of the Q wave to the end of the T wave) [3]–[5]. Much of this success has relied upon increasing sample size through meta-analyses across multiple cohorts, rather than through the use of novel analytical methods to increase power.

One analytical approach, gene-based tests proposed during the initial development of GWAS [6], has natural appeal. First, variations in protein-coding and adjacent regulatory regions are more likely to have functional relevance. Second, gene-based tests allow for direct comparison between different populations, despite the potential for different linkage disequilibrium (LD) patterns and/or functional alleles. Third, these analyses can account for multiple independent functional variants within a gene, with the potential to greatly increase the power to identify disease/trait-associated genes.

Despite these appealing properties, gene-based and related multi-marker association tests have generally under-performed single-locus tests when assessed with real data [7], [8]. A general drawback of methods that attempt to exploit the structure of LD to reduce the number of tests, for example through principal component analysis, is the loss of power to detect low-frequency alleles. Methods that consider multiple independent effects often require that the number of effects be pre-specified [9], which loses power when the tested and true model are different.

Multi-locus tests often have the additional practical drawback of being highly CPU and memory intensive. Several methods use Bayesian statistics to drive a brute-force sum or Monte Carlo sample over models [10], [11], but again often restrict the search to one or two-marker associations. In general, the computational costs have made these approaches infeasible for genome-wide applications.

The Gene-Wide Significance (GWiS) test addresses these problems by performing model selection simultaneously with parameter estimation and significance testing in a computational framework that is feasible for genome-wide SNP data (see Methods). Model selection, defined as identifying the best tagging SNP for each independent effect within a gene, uses the Bayesian model likelihood as the test statistic [12]–[14]. Our innovation is to use gene regions to impose a structured search through locally optimal models, which is computationally efficient and matches the biological intuition that the presence of one causal variant within a gene increases the likelihood of additional causal effects. Models are penalized based on the effective number of independent SNPs within a gene and the number of SNPs in the model, akin to a multiple-testing correction. The Schwarzian Bayesian Information Criterion corrects for the difference between the full model likelihood and the easily computed maximum likelihood estimate [15]. This method has greater power than current methods for genome-wide association studies and provides a principled alternative to *ad hoc* follow-up analyses to identify additional independent association signals in loci with genome-wide significant primary associations.

## Results

### Reference genotype and phenotype data

The ECG parameters PR interval, QRS interval and QT interval are ideal test cases because recent large-scale GWAS studies have established known positive associations. These traits are all clinically relevant, with increased PR interval associated with increased risk of atrial fibrillation and stroke [16], and both increased QRS and QT intervals associated with mortality and sudden cardiac death [17]–[20]. We assessed the ability of standard methods and GWiS to rediscover these known positives using data from only the Atherosclerosis Risk in Communities (ARIC) cohort, which contributes 15% of the total sample size for QRS, 25% for PR, and 50% for QT (Table 1).

**Table 1:** Populations, genes, and SNPs used in this study.

	PR	QRS	QT
Individuals, published GWAS	28,517	47,797	15,842/13,685
Individuals, this study	7,076	7,250	7,771
Individuals, this study relative to published	25%	15%	49%/57%
Genes, total	25,251		
Genes, at least one SNP assigned	24,337		
SNPs, total	2,557,232		
SNPs, assigned to at least one gene	1,392,262		
SNPs, average per gene	72		
SNPs, median per gene	43		
Effective tests, average per gene	9.3		
Effective tests, median per gene	7.3		

For SNP assignment, gene regions are defined to include 20 kb flanking transcription boundaries.

The SNPs were assigned to genes based on the NCBI *Homo sapiens* genome build 35.1 reference assembly [21]. Gene boundaries were defined by the most 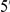 transcriptional start site and 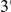 transcriptional end position for any transcript annotated to a gene, yielding 25,251 non-redundant transcribed gene regions. Incorporating additional flanking sequence increases coverage of more distant regulatory elements, which increases power, but also increases the number of SNPs tested, which decreases power. Expression quantitative trait loci (eQTL) mapping in humans has shown that most cis-regulatory SNPs are within 100 kb of the transcribed region [22], [23], with quantitative estimates that 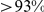 of large effect eQTNs (functional nucleotides that create eQTLs) are within 20 kb of the transcribed region [24]. We report results for 20 kb flanking regions; the performance ranking is robust to flanking by up to 100 kb (Table S1). SNPs within these regions are then assigned to one or more genes. Of the approximately 2.5 million genotyped and imputed SNPs, about 1.4 million are assigned to at least one gene. The median number of SNPs per gene is 43 and the mean is 72 (Table 1), reflecting a skewed distribution with many small genes having few SNPs.

The “gold standard” known positives rely on previously published meta-analyses of PR interval [1], QRS interval [2] and QT interval [4], [5]. We first identify gold-standard SNPs having 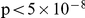. Any gene within 200 kb of a gold-standard SNP is classified as a known positive, and known positives within a 200 kb window are merged into a single locus, yielding 38 known positive gene-based loci. This procedure was followed to ensure that each association signal results in a single locus as opposed to being split between adjacent loci, which could result in over-counting.

### Other methods

The minSNP test uses the p-value for the best single SNP within a gene. The minSNP-P test converts this SNP-based p-value to a gene-based p-value by performing permutation tests within each gene. BIMBAM averages the Bayes Factors for subsets of SNPs within a gene, with restriction to single-SNP models recommended for genome-wide applications [10]. Because the Bayes Factor sum is dominated by the single best term, results for BIMBAM are very similar to minSNP-P. The Versatile Gene-Based Test for Genome-wide Association (VEGAS) [25] is a recent multivariate method that sums the association signal from all the SNPs within a gene and corrects the sum for LD to generate a test statistic. The 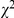 terms summed by VEGAS are asymptotically equivalent to the negative logarithms of the Bayes Factors summed by BIMBAM. LASSO regression, or *L*1 regularized regression, is a multivariate method that combines sparse model selection and parameter optimzation [26]–[28], with promising recent applications to GWAS [29]. See Methods for more details.

### Simulated data and power

**Figure 1:**
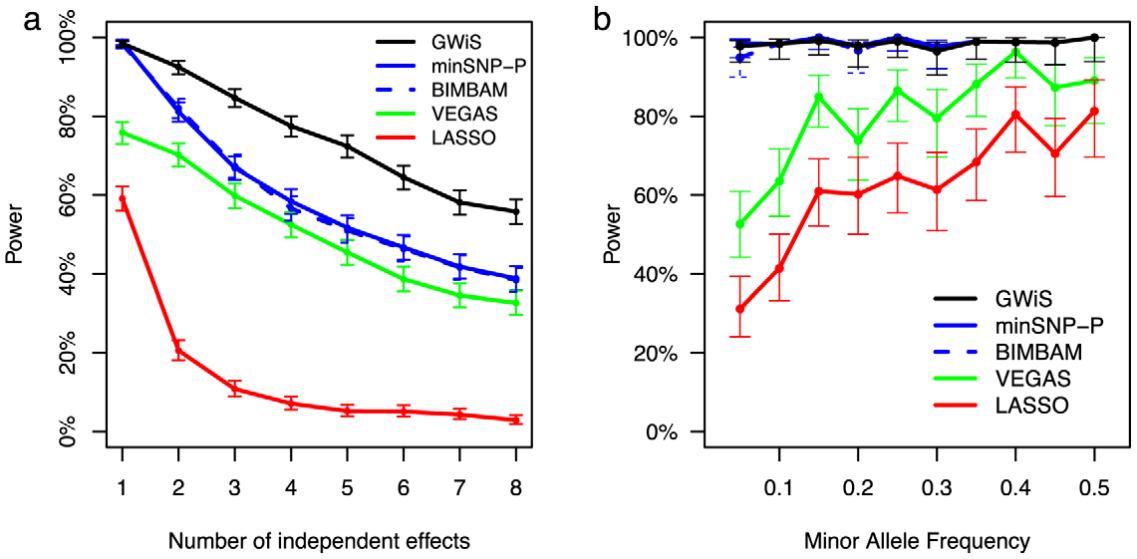
Estimated power at genome-wide significance for simulated data. Power estimates for GWiS (black), minSNP-P (blue), BIMBAM (dashed blue), VEGAS (green), and LASSO (red) are shown for 0.007 population variance explained by a gene. Genes were selected at random from Chr 1; genotypes were taken from ARIC; and phenotypes were simulated according to known models with up to 8 causal variants with independent effects. (a) Power decreases as total variance is diluted over an increasing number of causal variants. (b) Power estimates with 95% confidence intervals are shown as a function of minor allele frequency (MAF) for the simulations from panel (a) with a single independent effect. GWiS, minSNP, minSNP-P, and BIMBAM are robust to low minor allele frequency, whereas VEGAS and LASSO lose power.

Power calculations used genotypes from the ARIC population to ensure realistic LD. Phenotypes were then simulated for genetic models with one or more causal variants within a gene. GWiS was the best-performing method, with an advantage growing as more independent effects are present (Figure 1a). Theoretically, GWiS should have lower power than single-SNP tests when the true model is a single effect; according to the “no free lunch theorem”, this loss of power cannot be avoided [30]. The performance of GWiS therefore depends on the genetic architecture of a disease or trait: higher power if genes house multiple independent causal variants, and lower power if each gene has only a single causal variant. In practice, the loss of power was so slight as to be virtually undetectable.

Of the other methods, minSNP-P and BIMBAM had similar performance that degraded as the true model included more SNPs. The VEGAS test did not perform well, presumably because the sum over all SNPs creates a bias to find causal variants in LD blocks represented by many SNPs and to miss variants in LD blocks with few SNPs. In the absence of LD, with genotypes and phenotype simulated using PLINK [31], VEGAS performs better (Figure S1). The LASSO method performed worst.

The advantage of GWiS arises in part from better power to detect associations with low-frequency alleles (Figure 1b). GWiS, minSNP-P, and BIMBAM have roughly constant power for a given variance explained, regardless of minor allele frequency. In contrast, both VEGAS and LASSO suffer from a two-fold loss of power when minor allele frequencies drop from 50% to 5%. VEGAS may lose power because these low-frequency SNPs lack correlation with other SNPs, reducing the contribution to the VEGAS sum statistic. The LASSO penalty shrinks the regression coefficient, which may adversely affect SNPs with large regression coefficients that balance low minor allele frequencies.

### Simulated data and model size

**Figure 2:**
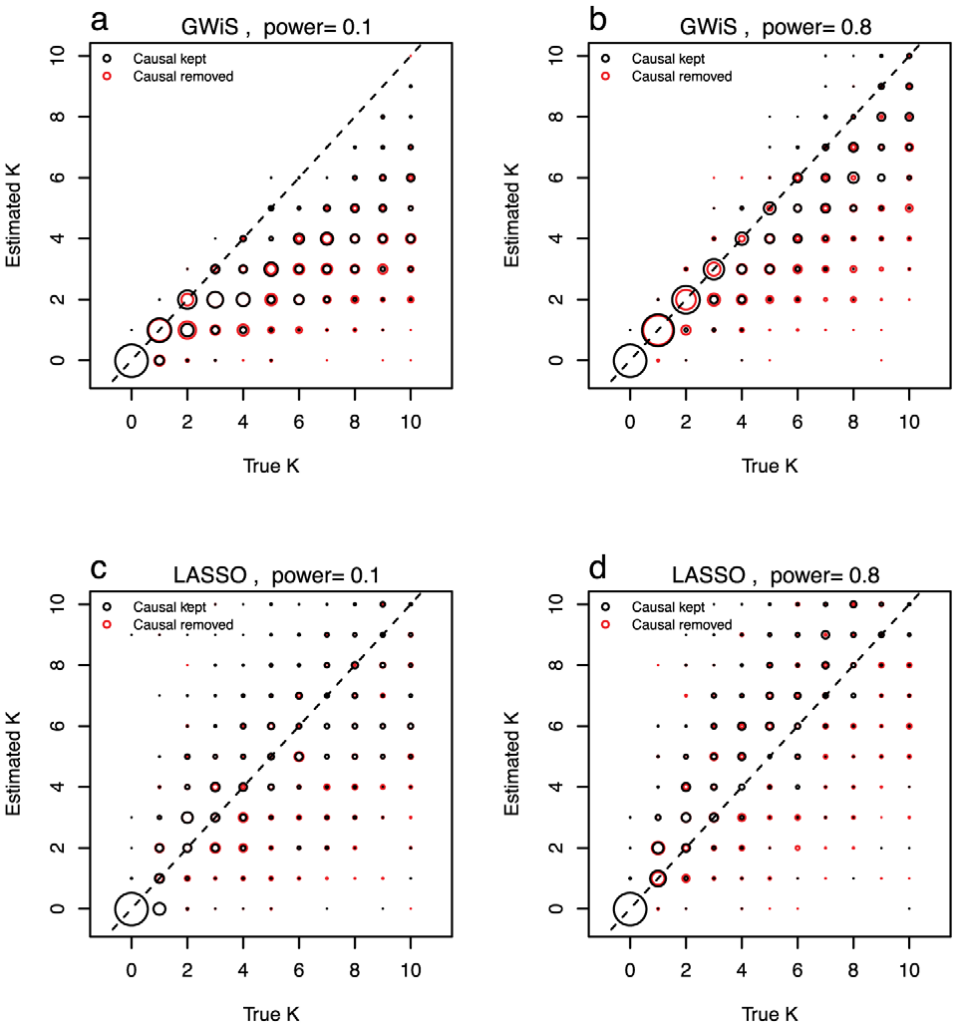
Model size estimation. The ability to recover the known model size was evaluated for GWiS (a and b) and LASSO (c and d). The power to detect a single SNP was set to be 10% (a and c) and 80% (b and d). In separate tests, the causal SNPs were either retained in (black) or removed from (red) the genotype data.

The model size selected by GWiS and LASSO was evaluated by simulation (Figure 2). These simulations also used the ARIC population to supply realistic LD, with genes selected at random with replacement from chromosome 1. In chromosome 1, the number of SNPs in a gene ranges from 1 to over 1000, and the number of independent effects ranges from 1 to over 100, similar to the distributions in the genome as a whole (Figure S2). A subset of SNPs within a gene had causal effects assigned (“True 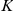
”), phenotypes were simulated to mimic weak and strong gene-based signals, and then models were selected by GWiS and LASSO. Model selection to retain a subset of SNPs (“Estimated 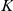
”) was performed both for the full genotype data and for the genotype data with the causal SNPs all removed.

GWiS provides a better estimate of the true model size than LASSO, assessed from the 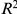 of estimated versus true 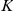. With causal SNPs kept, 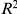 for GWiS is substantially higher, 0.65 versus 0.47 at low power (Figure 2a, 2c) and 0.81 versus 0.60 at high power (Figure 2b, 2d). GWiS also performs better when causal SNPs are removed, 0.55 versus 0.33 at low power and 0.60 versus 0.39 at high power. GWiS also provides a conservative estimate of the model size, with the ratio of estimated to true size ranging from a worst-case of 44% (low power, causal SNPs removed) to a best-case of 81% (high power, causal SNPs kept) over the four scenarios examined. In contrast, LASSO is prone to over-predict the size of the model, with a worst-case of models that are on average 33% too large (high power, causal SNPs kept, Figure 2d).

Removing a causal SNP results in GWiS predicting a smaller model, with the ratio of estimated to true 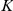 dropping from 0.55 to 0.44 for low power and from 0.85 to 0.81 for high power. These reductions in model size are highly significant (p 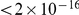 for both, Wilcoxon pair test) and counter a concern that the absence of a causal variant from a marker set will inflate the model size by introducing multiple markers that are partially correlated with the untyped causal variant.

These results demonstrate that the model size returned by GWiS is conservative for causal variants with small effects, and approaches the true model size for causal variants with large effects.

### Application to ECG data

We then obtained p-values from GWiS, minSNP, minSNP-P, BIMBAM, VEGAS, and LASSO for the ARIC data. Permutations of phenotype data holding genotypes fixed [32] provided thresholds for genome-wide significance for each method (Table S2). Due to LD across genes, a strong signal in one gene can lead to a neighboring gene reaching genome-wide significance. This effect is well known, and scoring these as false positives would unduly penalize traditional univariate tests. Instead, neighboring genes reaching genome-wide significance were merged, and overlap (even partial) with a known positive was scored as a true positive.

**Figure 3:**
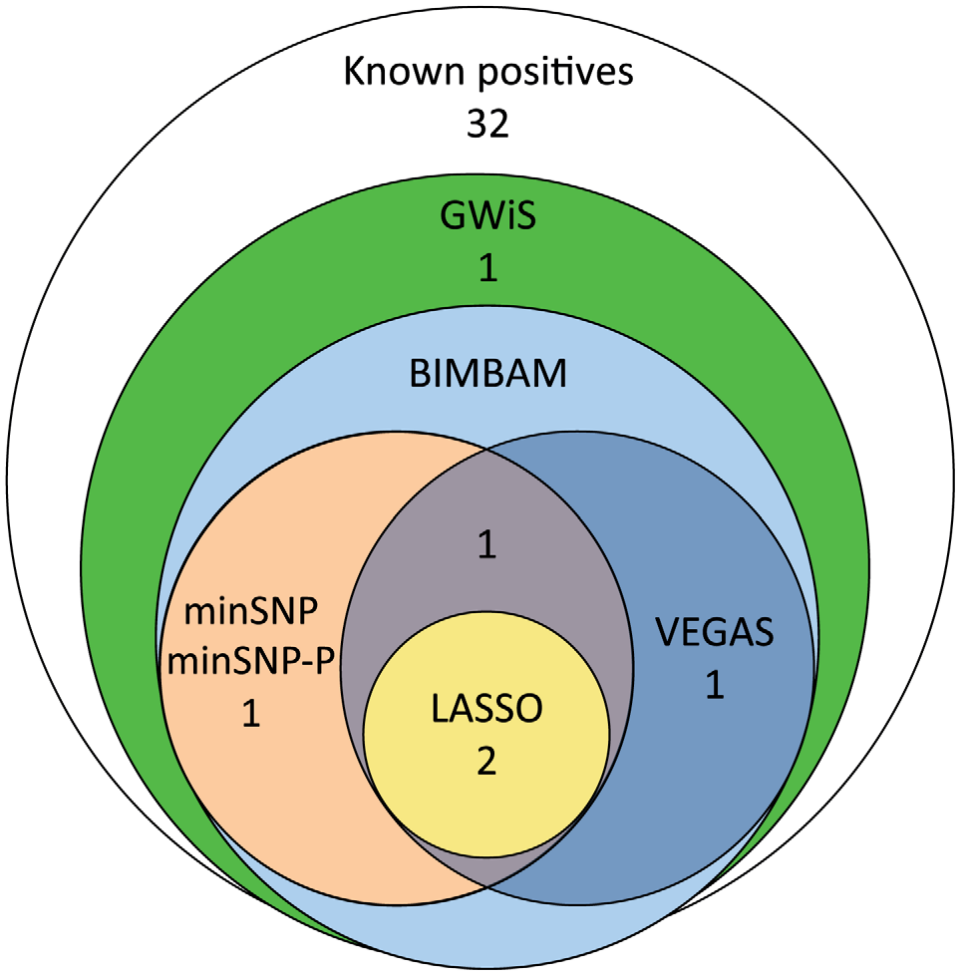
Recovery of known positive associations at genome-wide significance. Of 38 known positives, GWiS identified 6 at genome-wide significance with no false positives. Univariate methods (minSNP and minSNP-P) and VEGAS identified a subset of 4 entirely contained by GWiS, and LASSO identified a smaller subset of 2.

GWiS out-performed all other methods in the comparison (Figure 3 and Table 2). GWiS identifies 6 of 38 known genes or loci as genome-wide significant. In contrast, BIMBAM identifies 5 known positives; minSNP, minSNP-P and VEGAS identify 4; and LASSO identifies 2. Loci identified by the other methods are all subsets of the 6 found by GWiS. None of the methods produced any false positives at genome-wide significance.

**Table 2:** Recovery of known associations.

	Known Locus						GWiS (2E-6)						minSNP (7.4E-8)		minSNP-P (3E-6)		BIMBAM (3E-6)		VEGAS (1E-6)		LASSO (2.1E-11)			
Trait	Locus Name	Chr	Start	End	Genes	p-value	Genes	SNPs	Tests	*K*	p-value	Rank	p-value	Rank	p-value	Rank	p-value	Rank	p-value	Rank	Genes	*K*	SI	Rank
PR	SCN5A-SCN10A	3	38,363,244	39,055,168	8	2.1E-74	**2**	**259**	**32.7**	**6**	**8.2E-40**	**1**	**6.3E-24**	**1**	**1.1E-22**	**1**	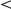 **1E-6**	**1**	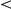 **1E-6**	**1**	**5**	**25**	**9.4E-33**	**1**
PR	CAV2-CAV1	7	115,444,532	116,032,391	4	3.7E-28	**1**	**93**	**7.8**	**1**	**2.1E-10**	**2**	**2.8E-11**	**2**	**2.1E-10**	**2**	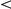 **1E-6**	**1**	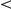 **1E-6**	**1**	2	3	1.3E-05	15
PR	ARHGAP24	4	87,208,605	87,281,000	1	6.2E-20	1	100	11.7				8.1E-03	1589	1.5E-01	3941	1.4E-01	3605	4.3E-02	581				
PR	SOX5-C12orf67	12	23,576,498	24,993,499	4	3.3E-13	1	185	15.8				8.9E-03	1664	4.5E-01	11033	2.8E-01	6964	5.2E-01	6092				
PR	ATP6VOE1-C5orf41-BNIP1-NKX2-5	5	172,264,849	172,687,936	9	9.5E-13	1	45	7.9	1	6.2E-05	5	2.0E-06	5	6.2E-05	5	2.6E-05	5	2.3E-06	3	5	6	5.4E-02	344
PR	MEIS1	2	66,574,183	66,711,542	1	4.6E-11	1	159	19.8				2.8E-04	156	2.5E-02	678	6.8E-03	138	3.7E-03	41	1	2	6.1E-04	163
PR	WNT11	11	75,203,923	75,783,791	5	3.2E-08	1	196	18.4	1	1.8E-03	46	3.1E-05	22	1.8E-03	48	2.5E-03	52	2.6E-01	3181	1	2	5.1E-05	35
QRS	ACVR2B-EXOG-SCN5A-SCN10A	3	38,322,431	39,055,168	9	1.1E-28	**4**	**318**	**34.6**	**4**	**3.4E-12**	**1**	1.0E-06	1	4.4E-05	1	4.0E-05	1	3.8E-06	1	4	11	1.8E-10	1
QRS	CDKN1A	6	36,518,522	37,000,310	11	3.0E-27	2	69	12.3	1	1.2E-04	4	5.0E-06	5	1.0E-04	3	8.7E-05	3	1.4E-03	14	2	3	8.2E-02	321
QRS	SLC35F1-PLN-BRD7P3	6	118,335,382	119,441,511	8	1.3E-18	1	31	6.8				4.1E-04	163	1.8E-02	338	2.5E-02	558	6.9E-03	62	1	1	4.5E-03	280
QRS	NFIA	1	61,260,314	61,634,057	1	4.6E-18	1	334	42.1	1	9.3E-03	138	1.1E-04	54	9.3E-03	139	2.2E-03	39	2.5E-03	21	1	2	2.1E-04	62
QRS	HAND1-SAP30L	5	153,550,488	153,845,903	5	7.4E-14	1	39	9.6				1.7E-03	515	1.3E-01	3157	1.5E-01	3770	1.6E-01	1963				
QRS	TBX20	7	34,982,033	35,189,326	3	1.1E-13	1	10	3.8				1.3E-03	417	1.5E-02	246	6.5E-03	105	5.5E-04	7				
QRS	SIPA1L1	14	70,443,875	71,275,875	5	1.0E-10	1	101	13.4				1.9E-03	558	1.6E-02	279	2.6E-02	567	8.2E-02	982				
QRS	TBX5	12	113,254,456	113,312,808	2	1.3E-10	1	133	19.1				1.2E-03	395	2.6E-02	536	1.8E-02	358	1.8E-02	198				
QRS	CDKN2C-FAF1	1	50,618,956	51,966,162	11	3.3E-10	1	56	11.2				6.6E-03	1317	5.9E-02	1433	3.7E-02	848	1.0E-01	1182				
QRS	GOSR2	17	41,805,904	42,621,664	8	4.8E-10	1	63	7.5	1	5.0E-03	84	3.9E-04	154	5.0E-03	85	1.7E-02	353	5.8E-02	703	2	2	3.7E-04	91
QRS	VTI1A	10	114,197,006	114,605,117	2	5.0E-10	1	271	32.2				6.9E-03	1342	1.1E-01	2782	9.8E-02	2411	2.0E-01	2344				
QRS	SETBP1	18	40,438,804	40,898,771	2	6.2E-10	1	98	11.5				1.1E-03	360	1.2E-01	2879	5.3E-02	1292	1.0E-02	93				
QRS	HEATR5B-STRN	2	36,835,565	37,370,391	8	1.9E-09	1	102	10.4	1	6.7E-03	103	4.5E-04	175	6.7E-03	102	1.5E-02	283	4.6E-02	548	2	2	4.0E-03	276
QRS	TKT-CACNA1D-PRKCD	3	53,099,055	53,356,653	5	6.3E-09	1	63	6.4				2.6E-02	5229	2.0E-01	4877	2.0E-01	4811	3.1E-01	3659				
QRS	CRIM1	2	36,495,048	36,736,886	2	8.2E-09	1	106	10.3				7.9E-04	287	6.0E-02	1464	5.2E-02	1252	8.9E-02	1072				
QRS	PRKCA	17	61,624,215	62,237,324	3	1.1E-08	1	611	53.4				3.6E-03	893	1.4E-01	3571	1.2E-01	2921	1.7E-01	2053				
QRS	LRIG1-SLC25A26	3	66,376,317	66,634,041	2	1.1E-08	1	144	14.8				1.7E-02	3353	2.1E-01	5157	3.0E-01	7296	3.4E-01	4002				
QRS	KLF12	13	73,158,150	73,606,043	1	1.3E-08	1	596	64.7				2.6E-04	121	5.5E-02	1338	5.6E-02	1351	6.2E-01	7218	1	3	1.7E-03	228
QRS	CASQ2	1	115,906,065	116,112,527	4	2.4E-08	1	189	13.8	1	2.5E-04	8	6.0E-06	7	2.2E-04	7	1.2E-04	4	6.1E-05	2	1	4	5.9E-01	333
QRS	DKK1	10	52,504,299	53,843,264	3	3.1E-08	1	22	5.6				6.2E-04	225	1.1E-01	2708	1.0E-01	2582	4.3E-01	5041				
QT	NOS1AP	1	158,467,768	159,113,560	6	1.9E-78	**1**	**506**	**41.3**	**2**	**2.2E-20**	**1**	**1.2E-18**	**1**	**4.9E-17**	**1**	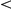 **1E-6**	**1**	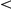 **1E-6**	**1**	**2**	**25**	**6.1E-25**	**1**
QT	GINS3-CNOT1	16	56,983,924	57,325,641	7	3.0E-25	1	101	12.6	1	4.2E-05	6	1.0E-06	7	4.2E-05	6	8.0E-06	4	9.7E-05	8	3	4	4.5E-05	42
QT	c6orf204-PLN	6	118,335,382	119,441,511	8	2.4E-24	**5**	**779**	**35.1**	**2**	**3.8E-10**	**2**	**6.2E-09**	**2**	**7.4E-08**	**2**	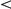 **1E-6**	**1**	4.2E-06	3	2	3	5.7E-02	326
QT	KCNQ1	11	2,246,305	2,826,916	6	2.8E-17	1	322	50.3	1	1.4E-03	37	2.0E-06	8	1.4E-03	36	6.5E-04	24	3.6E-03	43	1	2	1.4E-05	23
QT	RNF207	1	6,020,646	6,460,521	13	1.0E-16	5	20	6.7	1	5.0E-06	4	2.9E-07	4	5.0E-06	3	8.0E-06	4	1.8E-05	4	5	3	8.6E-06	19
QT	KCNH2	7	149,820,442	150,340,230	20	5.0E-16	**4**	**224**	**21.0**	**2**	**1.0-06**	**3**	6.9E-07	5	5.0E-06	3	**2.0E-06**	**3**	**1.0E-07**	**2**	5	6	5.3E-06	16
QT	ATP1B	1	165,633,513	166,331,065	8	1.2E-15	2	332	19.2	2	4.6E-05	7	1.2E-07	3	5.4E-05	7	2.2E-05	6	3.2E-03	36	4	6	5.2E-10	2
QT	LITAF	16	11,397,762	11,783,909	5	5.8E-15	3	236	34.6	2	5.8E-04	21	1.9E-05	20	5.7E-04	21	1.5E-03	32	9.6E-04	18	3	2	3.3E-07	7
QT	SCN5A	3	38,363,244	38,810,505	6	1.0E-14	2	148	21.3	1	1.8E-04	9	6.0E-06	13	1.8E-04	9	1.8E-04	12	1.7E-04	10	4	3	3.4E-06	12
QT	LIG3	17	30,279,055	30,618,866	11	6.0E-12	3	47	11.5	1	1.2E-05	5	1.0E-06	6	1.1E-05	5	2.6E-05	7	2.9E-05	5	5	5	1.7E-05	24
QT	KCNE1	21	34,658,193	34,909,252	5	2.0E-08	1	163	13.9				2.0E-02	4056	4.0E-01	9626	3.1E-01	7611	5.7E-01	6628				

The column “Genes” provides the number of genes in the locus; “SNPs” is the number of SNPs; “Tests” is the number of effective tests corrected for linkage disequilibrium within the locus; “*K*” is the number of SNPs in the model for GWiS and LASSO; and “SI” is the selection index for LASSO. The genome-wide significance threshold for each method is shown in parentheses next to the method name. The p-values for GWiS, minSNP-P, BIMBAM and VEGAS are gene-based; p-values for minSNP are SNP-based; and Selection Indices for LASSO are genome-wide. For each method, genome-wide significant findings are in **bold**. Blank entries for GWiS and LASSO indicate that no SNPs were added to the model for these loci. No SNPs within 200 kb of known loci for PR (TBX5-TBX3) and QT (KCNJ2) are genome-wide significant, and these loci are excluded from the table.

Due to the limited size of the ARIC cohort relative to the studies that generated the known positives, no method was expected to find all 38 known loci to be genome-wide significant. Nevertheless, known positives should still rank high among the top predictions of each method, assessed by the ranks of the known positives at 40% recall (Figure S3). We found that GWiS, minSNP, minSNP-P, BIMBAM, and VEGAS were equally effective in ranking known positives (Mann-Whitney rank sum p-values 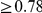 for any pairwise comparison). LASSO performed below the other methods (p-value 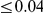 for a pairwise comparison of LASSO to any other method). Top associations (up to 100 false positives) from each method are provided for PR interval, QRS interval, and QT interval (Tables S3, S4, S5).

**Figure 4:**
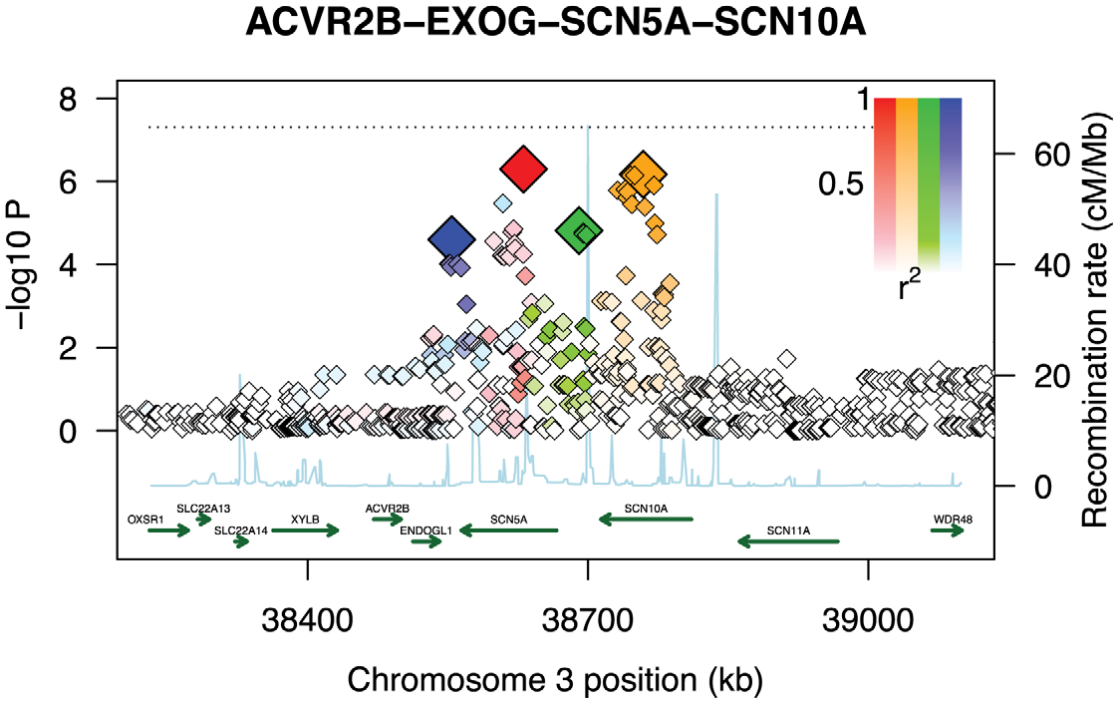
Multiple weak effects identified as genome-wide significant. GWiS correctly identifies the SCN5A-SCN10A locus as genome-wide significant with four independent effects, even though the strongest single effect has a p-value 100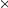 worse than the genome-wide significance threshold indicated as a dashed line. No other method was able to identify this locus as genome-wide significant. The SNPs selected by GWiS are represented as large, colored diamonds, and SNPs in LD with these four are colored in lighter shades. The light blue trace indicates recombination hotspots.

While our conclusions are based on cardiovascular phenotypes, the results suggest that GWiS will have an advantage when causal genes have multiple effects. When an association is sufficiently strong to be found by a univariate test, GWiS is generally able to identify it. Beyond these association, GWiS is also able to detect genes that are genome-wide significant, but where no single effect is large enough to be significant by univariate tests. The association of QRS interval with SCN5A-SCN10A is a striking example: 4 independent effects are found by GWiS (p-value = 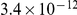) but the association is not genome-wide significant by univariate methods (p-value = 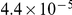 for minSNP-P) (Figure 4). A common follow-up strategy for single-SNP methods is to search for secondary associations in the same locus as a strong primary association. These results for ARIC together with results above for simulated data (Figure 2) demonstrate that GWiS performs this task well. While this feature is present in previous follow-up methods for candidate loci [11], [33], [34], it is absent from methods generally used for primary analysis of GWAS data.

**Figure 5:**
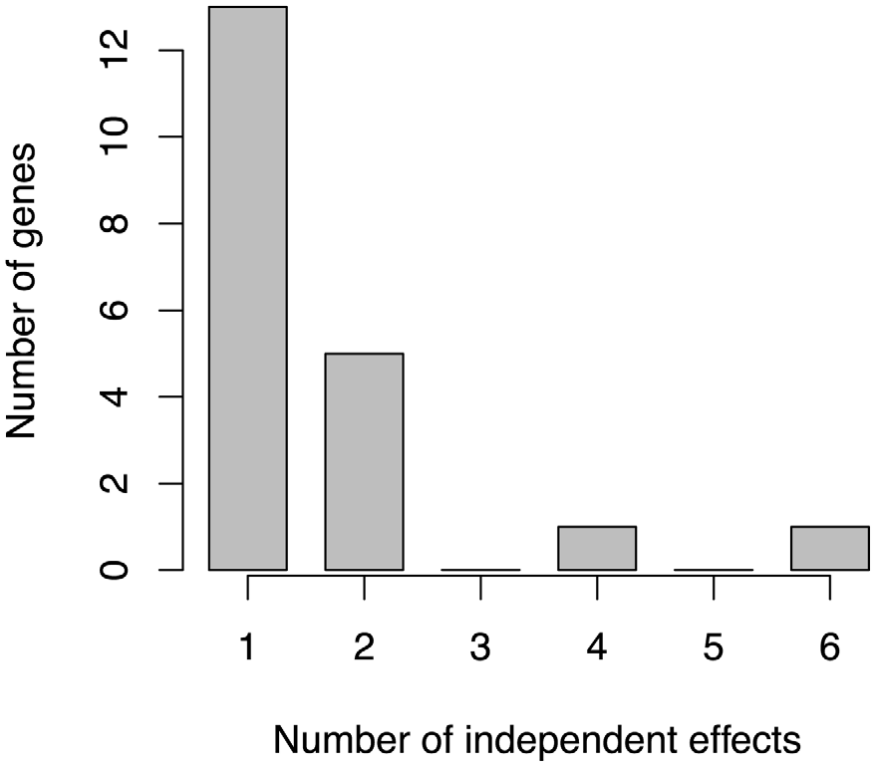
Distribution of the number of independent effects in ECG loci. Of 38 known positive loci, GWiS identified 20 loci, and 7 of these contain multiple independent effects.

Of the 38 known positives, 20 have GWiS models with at least one SNP (regardless of genome-wide significance), and 7 of these are predicted to have multiple independent effects (Figure 5). These results suggest that the genetic architecture of ECG traits supports the hypothesis underlying GWiS. Moreover, for QT interval where the power is greatest to identify known positives (the ARIC sample size is 50% of the GWAS discovery cohorts), 5 of the 10 loci identified by GWiS are predicted to have multiple independent effects.

## Discussion

In summary, we describe a new method for gene-based tests of association. By gathering multiple independent effects into a single test, GWiS has greater power than conventional tests to identify genes with multiple causal variants. GWiS also retains power for low-frequency minor alleles that are increasingly important for personal genetics, a feature not shared by other multi-SNP tests.

Furthermore, GWiS provides an accurate, conservative estimate for the number of independent effects within a gene or region. Currently there are no standard criteria for establishing the genome-wide significance of a weak second association in a gene whose strongest effect is genome-wide significant. While the number of effects can be provided by existing Bayesian methods [34], their computational expense has limited their applicability to candidate regions, and they are not routinely used. By providing a computationally efficient alternative to existing methods, GWiS provides a new capability to estimate the number of effects as part of primary GWAS data analysis. Demonstrated effectiveness on real data may lead to more widespread use of this type of analysis. Applied to cardiovascular phenotypes relevant to sudden cardiac death and atrial fibrillation, GWiS indicates that 35 to 50% of all known loci contain multiple independent genetic effects.

The test we describe includes a prior on models designed to be unaffected by SNP density, in particular by the number of SNPs that are well-correlated with a causal variant. The priors on regression parameters are essentially uniform, with the benefit of eliminating any user-adjustable parameters. A theoretical drawback is that the priors are improper [35], [36]. Theoretical concerns are mitigated, however, because improper priors pose no challenge for model selection, and our permutation procedure ensures uniform p-values under the null.

Bayesian methods can be computationally expensive. GWiS minimizes computation by evaluating only the locally optimal models of increasing size in a greedy forward search. This appears to be an approximation compared to previous Bayesian methods that sum over all models. Previous Bayesian methods entail their own approximations, however, because the search space must either be truncated at 1 or 2 SNPs, heavily pruned, or lightly sampled using Monte Carlo. Our results demonstrate that the approximations used by GWiS provide greater computational efficiency than approximations used in previous Bayesian frameworks, with no loss of statistical power. GWiS currently calculates p-values, rather than Bayesian evidence provided by other Bayesian methods. If Bayesian evidence is desired, an intriguing alternative to Bayesian post-processing of candidate loci might be to use the Bayes Factor from the most likely alternative model identified by GWiS as a proxy for the sum over all alternatives to the null model. This may be an accurate approximation because, in practice, the Bayes Factor for the most likely model from GWiS dominates all other Bayes Factors in the sum.

The GWiS framework, using gene annotations to structure Bayesian model selection, may be applied to case-control data by encoding phenotypes as 1 (case) versus 0 (control), a reasonable approach when effects are small. More fundamental extensions to logistic regression, Transmission Disequilibrium Tests (TDTs), and other tests and designs should be possible and may yield further improvements. Moreover, similar gene-based structured searches can be applied to genetic models to include explicit interaction terms [14]. The Bayesian format also permits incorporation of prior information about the possible functional effects of SNPs [37], [38], and disease linkage [39], [40]. Finally, the gene-based p-values provide a natural entry to gene annotations and pathway-based gene set enrichment analysis [41]–[43].

## Materials and Methods

### Ethics statement

This research involves only the study of existing data with information recorded in such a manner that the subjects cannot be identified directly or through identifiers linked to the subjects.

### Known positives

Known positive associations are taken from published genome-wide significant SNP associations (p-value 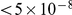) [1], [2], [4], [5]. Genes within 200 kb of any genome-wide significant SNP are scored as known positives. Finally, genes within 200 kb that are both positive are merged into a single known positive locus to avoid over-counting.

### Study cohort

The ARIC study includes 15,792 men and women from four communities in the US (Jackson, Mississippi; Forsyth County, North Carolina; Washington County, Maryland; suburbs of Minneapolis, Minnesota) enrolled in 1987-89 and prospectively followed [44]. ECGs were recorded using MAC PC ECG machines (Marquette Electronics, Milwaukee, Wisconsin) and initially processed by the Dalhousie ECG program in a central laboratory at the EPICORE Center (University of Alberta, Edmonton, Alberta, Canada) but during later phases of the study using the GE Marquette 12-SL program (2001 version) (GE Marquette, Milwaukee, Wisconsin) at the EPICARE Center (Wake Forest University, Winston-Salem, North Carolina). All ECGs were visually inspected for technical errors and inadequate quality. Genotype data sets were cleaned initially by discarding SNPs with Hardy-Weinberg equilibrium violations at p 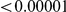, minor allele frequencies 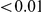, or call rates 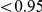. Imputation with HapMap CEU reference panel version 22 was then performed, and all imputed SNPs were retained for analysis, included imputed SNPs with minor-allele frequencies as low as 0.001. These cleaned data sets contributed to the meta-analysis to yield the known positives, and full descriptions of phenotype and sample data cleaning are available elsewhere [1], [2], [4]. Regional association plots were generated using a modified version of “make.fancy.locus.plot” [45].

### Conventional multiple regression

The phenotype vector **Y** for *N* individuals is an 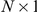 vector of trait values. The genotype matrix **X** has *N* rows and *P* columns, one for each of *P* genotyped markers assumed to be biallelic SNPs. For simplicity, the vector **Y** and each column of **X** are standardized to have zero mean. A standard regression model estimates the phenotype vector as 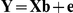, where **b** is a vector of regression coefficients and **e** is a vector of residuals assumed to be independent and normally distributed with mean 0 and variance 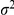 . The log probability of the phenotypes given these parameters is
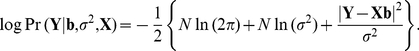
(1)


The maximum likelihood estimators (MLEs) are 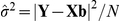 and 
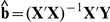
, where 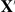 denotes the transpose of 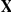. The total sum-of-squares (SST) is 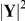, and the sum-of-squares of the model (SSM) is 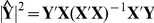 . The sum-of-squares of the errors or residuals (SSE) is
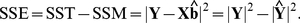
(2)


A conventional multiple regression approach uses the *F*-statistic to decide whether adding a new SNP improves the model significantly,
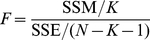
(3)for a model with *K* SNPs, distributed as 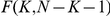 under the null. This approach fails, however, when the best 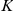 SNPs are selected from the much larger number of *M* total SNPs, because the 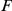 statistic does not account for the selection process.

### Bayesian model selection

A model *M* is defined as the subset of 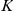 SNPs in a gene with *P* total SNPs that are permitted to have non-zero regression coefficients. For each gene, GWiS attempts to find the subset that maximizes the model probability 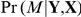, where each of the *P* columns of **X** corresponds to a SNP assigned to the gene. In the absence of association, the null model with 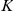 = 0 usually maximizes the probability, indicating no association. When a model with 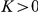 maximizes the probability, an association is possible, and permutation tests provide a p-value. According to Bayes rule,
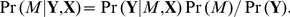
(4)


The factor 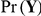 is model-independent and can be ignored.

The prior probability of the model, 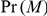, assumes that each of the *P* SNPs within the gene has an identical probability of being associated with the trait. This probability, denoted *f*, is unknown, and is integrated out with a uniform prior. The prior is also designed to make the model probability insensitive to SNP density: it should be unaffected if an existing SNP is replicated to create a new SNP marker with identical genotypes. We do this by replacing the number of SNPs within a gene with an effective number of tests, 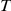, calculated from the local LD within a gene. Correlations between SNPs make the effective number of tests smaller than the number of SNPs. The model prior based on the effective number of tests is
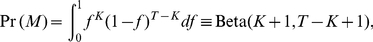
(5)or 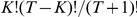 for integer values. As the effective number of tests, 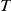, whose calculation is described below, is generally non-integer, we use the standard Beta function rather than factorials.

The remaining factor in Eq. 4 is
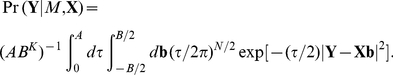
(6)


The integration limits and prefactor 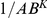 ensure normalization. We assume that these limits are sufficiently large to permit a steepest descents approximation as in Schwarzian BIC model selection [15]. First, assuming that the genotypes are centered, the genotype covariance matrix is 
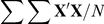
, where 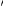 indicates matrix transpose as before, and diagonal elements 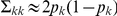 for SNP 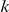 with allele frequency 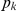. Provided that 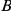 is much greater than each component of 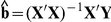, the integral over 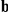 is approximately
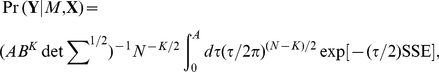
(7)where the sum-squared-error SSE is 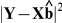. Provided that the limit 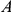 is much greater than the maximum likelihood value 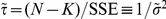, the integral over 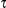 can be approximated as
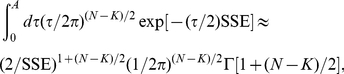
(8)where 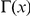 is the standard Gamma function. To avoid the cost of Gamma function evaluations, we instead use the steepest descents approximation,
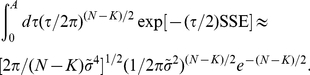
(9)


The log-likelihood is then
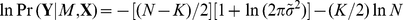


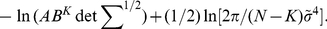
(10)


As in the BIC approximation, we retain only terms that depend on the model and are of order 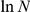 or greater. Thus we replace 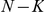 by 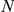, and 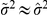. For historical reasons, we also included a factor of 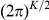 in the prior for model size, to yield the asymptotic approximation
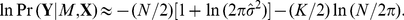
(11)


The strategy of GWiS is therefore to find the model that maximizes the objective function
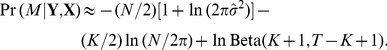
(12)


The terms involving 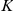 provide a Bayesian penalty for model performance, but also make this an NP-hard optimization problem. We have adopted two efficient deterministic heuristics for approximate optimization. First is a greedy forward search, essentially Bayesian regularized forward regression, in which the SNP giving the maximal increase to the model likelihood is added to the model sequentially until all remaining SNPs decrease the likelihood. The second is a similar heuristic, except that the initial model searches through all subsets of 2 SNPs or 3 SNPs. We adopted this subset search to permit the possibility that all 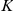 = 1 models are worse than the 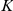 = 0 null, whereas a more complex model with 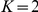 or 3 has higher score. In practice, all associations identified by subset selection were also identified by greedy forward search. We therefore used the greedy forward search for computational efficiency.

GWiS is designed to select a single model for each gene. An alternative related approach would be to test for the posterior probability of the null model, 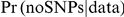, against all other models, 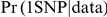 + 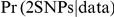 + 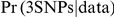 + 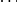, using our model selection procedure either to choose the locally best model of each size or to include multiple models (which could suffer from a systematic bias favoring SNPs in large LD blocks). This is in fact the strategy of BIMBAM, which attempts to systematically evaluate all terms up to a given model size. Unfortunately, the number of terms increases exponentially fast with model size, and the brute-force approach does not scale to genome-wide applications. Monte Carlo searches over models have also been difficult to apply genome-wide. Our work suggests that approximations that limit the search for fixed model size can be accurate, and further that the probabilities of models that are too large are expected to decrease exponentially fast, permitting the sum to be pruned and truncated. We have observed in practice that the model with the most likely value of 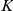 dominates the sum, and similarly for BIMBAM that the single SNP with the best Bayes Factor dominates the sum-of-Bayes-Factors test statistic. These results suggest that the results of a more computationally expensive sum over all models would be largely consistent with the results of GWiS method. Furthermore, the Bayes Factor for the most likely model could provide a proxy for the Bayesian evidence.

### Effective number of tests

The effective number of tests is an established concept in GWAS to provide a multiple-testing correction for correlated markers. While the exact correction can be established by permutation tests, faster approximate methods can perform well [46]–[49]. While we use a fast procedure, a final permutation test ensures that p-values are uniform under the null.

The method we adopt is based on multiple linear regression of SNPs on SNPs. The genotype vector 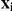 for each SNP *i* is standardized to have zero mean. Correlations between all pairs of SNPs *i* and *j* are initialized as 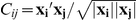. Each SNPs weight 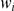 is initialized to 1, and the number of effective tests *T* is initialized to 0. The SNP *i* with maximum weight is identified, and the following updates are executed:
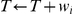


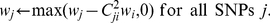
(13)


This process continues until all weights are equal to zero. When SNPs with maximum weight are tied (as occurs for the first SNP processed), the SNP with lowest genomic coordinate is selected to ensure reproducibility; we have ensured that this method is robust to other methods for breaking ties, including random selection. For simplicity, the correlations are not updated (the update rule would be 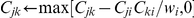), which may lead to an overestimate for *T*. Model selection may therefore have a conservative bias. The p-values are not affected, however, because they are calculated by permutation tests as described below.

The effective number of tests implies a trivial renormalization of the model prior, (Eq. 5), that does not affect the test statistic. Letting 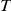 be the total number of markers, 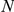 be the effective number tests, and 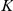 be the size of the model, our prior gives each model of size 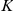 the weight 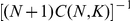. If 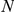 and 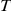 are identical, there are 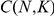 models of this size, and the total weight of all models of size 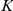 is 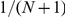. Since 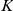 can range from 0 to 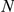, the sum is normalized. But when 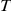 is larger than 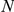, the sum of all models of size 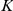 is 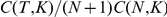, which is 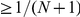. The sum from 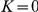 to 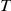 is therefore 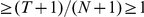. A normalization of 1 can be recovered by including an overall normalization factor, 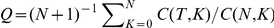. The explicit prior for models of size 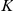 is 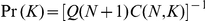, which is normalized to 1. Since 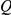 is model-independent, it does not contribute to the test statistic.

### P-values and genome-wide significance

We use two stages of permutation tests: the first stage converts the GWiS test statistic into a p-value that is uniform under the null; the second stage establishes the p-value threshold for genome-wide significance.

The first stage is conducted gene-by-gene. We permute the trait array using the Fisher-Yates shuffle algorithm [50], [51] and use the permuted trait to calculate the test statistics using the same procedure as for the original trait. Specifically, the model size 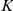 is optimized independently for each permutation, with most permutations correctly choosing 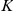 = 0. For *S* successes (log-likelihoods greater than or equal to the unpermuted phenotype data) out of *Q* permutations, the empirical p-value is *S*/*Q*. To save computation, permutations are ended when 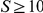. Furthermore, once a finding is genome-wide significant, there is no practical need for additional permutations. For gene-based tests (GWiS, minSNP-P, BIMBAM, and VEGAS), the p-value for genome-wide significance depends on the number of genes tested (rather than the number of SNPs), 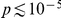 for humans. We therefore also terminate permutations after *Q* = 1 million trials, regardless of *S*. In these cases, for purposes of ranking, a parametric p-value is estimated for GWiS as
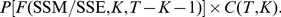
(14)


The first factor is the parametric p-value for the *F* statistic from the MLE fit, and the second term is the combinatorial factor for the number of possible models of the same size.

While these p-values are uniform under the null, the threshold for genome-wide significance requires a second set of permutations. To establish genome-wide significance thresholds, in the second stage we permuted the ARIC phenotype for each trait 100 times, ran GWiS for the permuted phenotypes on the entire genome, and recorded the best genome-wide p-value from each of the 100 permutations. We then combined the results from each trait to obtain an empirical distribution of the best genome-wide p-value under the null. We then estimated the p = 0.05 genome-wide significance threshold as the 15th best p-value of the 300. This procedure was performed for GWiS, minSNP, minSNP-P, LASSO, and VEGAS to obtain genome-wide significance thresholds for each. Since minSNP-P and BIMBAM are both uniform under the null, we used the genome-wide significance threshold calculated for minSNP-P, 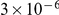, for BIMBAM to avoid additional computional cost (Table S2). The threshold for GWiS is more stringent, 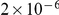, presumably because of the locus merging procedure described below. Changes in the genome-wide significance thresholds of up to 50% would not affect any of the reported results.

### Hierarchical analysis of genetic loci

In a region with a strong association and LD, GWiS can generate significant p-values for multiple genes in a region. A hierarchical version of GWiS is used to distinguish between two possibilities. First, through LD, a strong association in one gene may cause a weaker association signal in a second gene. In this case, only the strong association should be reported. Second, the causal variant may not be localized in a single gene; for example, the best SNP tags are assigned to multiple genes. In this case, the individual genes should be merged into a single associated locus. The hierarchical procedure is as follows.

1. Identify all genes with GWiS 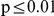, and use transitive clustering to merge into a locus all genes whose transcript boundaries are within 200 kb.
2. Run GWiS on the merged locus (including a recalculation of the number of effective tests within the locus) and identify the SNPs selected by the GWiS model. If genes at either end of the locus have no GWiS SNPs, trim these genes from the locus. Repeat this step until no more trimming is possible. If only a single gene remains, accept it with its original p-value as the only association in the region. Otherwise, proceed to step 3.
3. Use a permutation test to calculate the p-value for the merged locus from step 2. Assign it a p-value equal to the minimum of the p-values from the individual genes, and the p-value from its own permutation. Regardless of the p-value used, retain the entire trimmed region as an associated locus.

The trimming in step 2 handles the first possibility, a strong association in one gene that causes a weaker association in a neighbor. The rationale for accepting the smallest p-value in step 3 is the case of a single SNP assigned to multiple genes. The merged region will have a less significant p-value than any single gene, and it does not seem reasonable to incur such a drastic penalty for gene overlap.

### Univariate tests: minSNP and minSNP-P

For these tests, SNPs are assigned to gene regions as before. The p-value for each SNP is then calculated using the *F*-statistic as the test statistic, with empirical p-values from permutation to ensure correct p-values for SNPs with low minor allele frequencies. The minSNP method assigns a gene the p-value of its best SNP. Selection of the best p-value out of many leads to non-uniform p-values under the null. It is standard to reduce this bias by scaling p-values by a Bonferroni correction based on the number of SNPs or number of estimated tests. Instead, we perform gene-by-gene permutation tests using the best *F* statistic for SNPs within the gene as the test statistic. As with GWiS, if 1 million permutations do not lead to one success, the association is clearly genome-wide significant and we use the Bonferroni-corrected p-value for ranking purposes.

### BIMBAM

The Bayesian Imputation-based Association Mapping (BIMBAM) is a Bayesian gene-based method [10]. BIMBAM calculates the Bayes Factor for a model and then averages the Bayes Factors for all models within a gene to obtain a test statistic. Because 1-SNP models were found to have as much power as 2-SNP models, and because 2-SNP models are not computationally feasible for genome-wide analysis, BIMBAM by default restricts its sum to all 1-SNP models within a gene [10]. The Bayes Factor 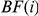 for a single SNP 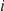 is
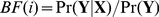
(15)

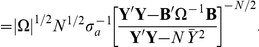



The design matrix 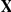 has first column 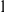
s and second column equal to the dosages of SNP 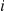 in the 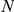 individuals; 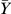 is the phenotypic mean; 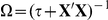; the matrix 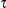 is diagonal with diagonal terms 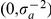; and 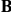 contains the regression coefficients 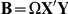. We used the recommended value 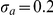 relative to the phenotypic standard deviation. The test statistic for a gene with 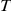 SNPs is 
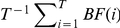
. As with other methods, we used gene-by-gene permutations to convert this statistic into a p-value that is uniform under the null. Up to 1 million permutations were used, stopping after 10 successes.

The sufficient statistics used by BIMBAM are identical to minSNP and minSNP-P, yet we found that the runtime of the public implementation was much slower, taking 270 sec for 1000 permutations of a gene with 135 SNPs across 8000 individuals. By improving memory management and optimizing computations, we improved the timing to 14 sec per 1000 permutations, a 19-fold speed-up. This implementation is included in our Supplementary Materials.

### VEGAS

The Versatile Gene-Based Test for Genome-wide Association (VEGAS) [25] is a recently proposed method that considers the SNPs within a gene as candidates for association study. VEGAS assigns SNPs to each of the autosomal genes using the UCSC genome browser hg18 assembly. The gene boundaries are defined as 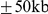 of the 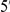 and 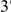 UTRs. Single SNP p-values are used to compute a gene-based 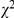 test statistic for each gene and significance of each gene is evaluated using simulations from a multivariate normal distribution with mean 0 and covariance matrix being the pairwise LD values between the SNPs from HapMap Phase 2. As a result the method avoids permutations in calculating per gene p-values, although permutations are required to obtain the genome-wide significance threshold.

### LASSO regression

LASSO regression is a recent method for combined model selection and parameter estimation that maps *L*1 regularized regression onto a computationally tractable quadratic optimization problem [26]–[28]. Applications to GWAS are attractive because it is possible to perform model selection on an entire chromosome. We therefore implemented a recent LASSO procedure developed specifically for GWAS [29].

To reduce computational cost, univariate p-values are estimated from parametric tests, and gene-based SNPs with 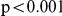 are retained (we have confirmed that this computational constraint does not lose any known positive associations). Incremental model selection was performed by Least Angle Regression [27] using the R lars package [52]. The LASSO parameter was determined using 5-fold cross validation. All genes with at least one SNP selected were identified, and selected genes overlapping other selected genes (including flanking regions) were merged into single loci.

As suggested previously, we used the Selection Index to rank genes and as the test statistic for a permutation p-value [29]. To obtain the Selection Index, the MLE log-likelihood is calculated for the full model and for a reduced model with a subset of SNPs removed. Twice the log-likelihood difference is interpreted as a 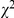 statistic, and the Selection Index is defined as the corresponding p-value for a 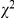 distribution with the number of removed SNPs as the degrees of freedom. Due to the LASSO model selection procedure, the Selection Index is not distributed as a 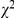 under the null, and permutation tests are used to establish genome-wide significance levels.

### Simulations: power

For each true model size of 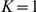 to 8, we performed a series of simulations by picking 1000 genes from chromosome 1 randomly with replacement, using genotype data from the ARIC population of approximately 8000 individuals. For each gene, we selected 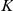 “causal” SNPs that have 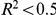 from regression with other “causal” SNPs within the gene. A gene had to have at least 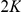 SNPs to be picked for models of size 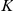 to ensure enough remaining SNPs after the removal of the causal SNPs to permit a model of the true size.

We attempted to distribute the total population variance explained, 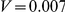, equally across the 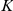 SNPs. The covariance matrix for the SNPs calculated from the population is denoted 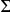, with 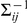 understood to be 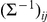. The coefficient 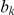 for SNP 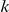 in the model was set to
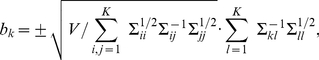
(16)which ensures that 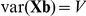. The phenotype 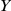 for an individual with genotype row-vector 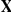 was then calculated as 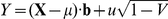, with 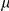 again the population average of 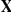 and 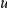 drawn from a standard normal distribution.

The power was calculated as (number of genes that are genome-wide significant)/1000, and the error of the estimate was calculated using 95% exact binomial confidence intervals. The p-value thresholds were taken directly from genome-wide permutations (Table 2).

### Simulations: model size

Phenotypes that were used to estimate the model size were generated by assigning each “causal” SNP the same power of 0.1 and 0.8. The population variance explained for each SNP was calculated as 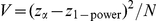, in which 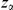 is the quantile of the standard normal for upper-tail cumulative probability of 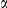, and 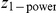 is the quantile for lower-tail probability 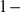
power. We chose 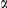 to be 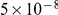, the commonly used genome-wide significance threshold for univariate tests. The effect of SNP 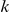 is then 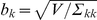, in which 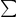 is the genotype covariance matrix. The simulated phenotypes are then 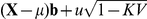, with 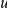 drawn from a standard normal distribution. In this test we control for the variance explained by the SNP, not by the gene, and therefore do not rescale the regression coefficients to account for LD. For each 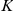 ranging from 0 to 10, we repeated these steps using ARIC genotype data for 100 genes chosen at random from chromosome 1.

Only GWiS and LASSO give model size estimates. GWiS directly reports the model size as the number of independent effects within a gene and LASSO reports the model size as the number of selected SNPs within a gene. We ran both methods using the simulated data with LD. We also tested both scenarios when the causal SNPs were kept or removed from gene.

### Performance evaluation

Gene associations were scored as true positives if the gene (or merged locus) overlapped with a known association, and as false positives if no overlap exists. Only the first hit to a known association spanning several genes was counted.

The primary evaluation criterion is the ability to identify known positive associations at genome-wide significance. The genome-wide significance threshold was determined separately for each method (see above), and no method gave any false positives at its appropriate threshold.

A secondary criterion was the ability to enrich highly ranked loci for known associations, regardless of genome-wide significance. This criterion was assessed through precision-recall curves, with precision  =  TP/(TP+FP), recall  =  TP/(TP+FN), and true positives (TP), false positives (FP), and false negatives (FN) defined as a function of the number of predictions considered.

Small differences in precision and recall may not be statistically significant. To estimate statistical significance, we performed a Mann-Whitney rank sum test for the ranks of the known associations at 40% recall for GWiS, minSNP, minSNP-P, and LASSO.

### Implementation

GWiS runs efficiently in memory and CPU time, roughly equivalent to other genome-wide tests that require permutations (Table 3). Computational times are greater for real data because real associations with small p-values require more permutations. LASSO required far less computational resources, but also pre-filtered the SNPs and had the worst performance. Genome-wide studies can be finished within around 100 hours. Low memory requirements allow GWiS to run in parallel on multiple CPUs. The GWiS source code implementing GWiS, minSNP, minSNP-P, and BIMBAM is available under an open source GNU General Public License as Supplementary Material, also from the authors' website (www.baderzone.org), and is being incorporated into PLINK [31].

**Table 3:** Memory and CPU requirements.

		Memory (GB)		CPU time (Hours)	
Method	Phenotype	Null	Real	Null	Real
GWiS	PR	1.2	1.2	9.4	43.1
	QRS	1.2	1.2	11.0	31.9
	QT	1.2	1.2	11.2	67.0
minSNP	PR	0.6	0.6	13.6	62.0
	QRS	0.6	0.6	15.8	45.9
	QT	0.6	0.6	16.1	96.4
minSNP-P	PR	0.6	0.6	11.9	54.2
	QRS	0.6	0.6	13.8	40.1
	QT	0.6	0.6	14.0	84.3
BIMBAM	PR	0.6	0.6	14.1	42.3
	QRS	0.6	0.6	16.5	33.2
	QT	0.6	0.6	16.8	101.5
VEGAS	PR	32.5	8.2	26.0	34.0
	QRS	26.0	11.9	23.9	29.8
	QT	25.8	14.1	27.1	33.0
LASSO	PR	0.1	0.1	0.2	0.4
	QRS	0.1	0.1	0.3	0.3
	QT	0.1	0.1	0.2	0.4

The minimal memory requirement and the total CPU time to finish one genome-wide study are reported for both a null (shuffled) trait and the real trait. Benchmarks were obtained from AMD Operon 2.3GHz or similar processors. The memory and CPU requirements include the model selection and the calculation of the gene-based p-values (or selection index). Costs for the genome-wide permutations to establish genome-wide significance thresholds are not included in the estimates. LASSO consumes the least resources because it pre-filters the SNPs (only uses SNPs having p-values 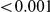) and does not require permutations to calculate the selection index. The real phenotypes require more CPU time because more permutations are required to calculate genome-wide significant p-values for true associations.

## Supporting Information

Figure S1Estimated power at genome-wide significance for genotypes simulated without LD. Simulation tests were performed for true models in which a single gene housed one to eight independent causal variants. Genotypes were simulated with 20 SNPs per gene, no LD between SNPs, and minor allele frequencies selected uniformly between 0.05 and 0.5. Power estimates are provided for VEGAS (green), GWiS (black), minSNP-P (blue), BimBam (blue dashed), and LASSO (red). While VEGAS performs well in the absence of LD, its performance degrades under realistic LD (see main text, Figure 1). We simulated genetic models for quantitative traits with no linkage disequilibrium between SNPs using the simulate-qt option of PLINK. Genes were simulated with 20 SNPs and minor allele frequencies selected uniformly between 0.05 and 0.5. Genotypes were coded as allele dosages from 0 to 2. The power of a standard regression test for additive effects depends on the population variance explained, 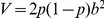 for a single variant with allele frequency 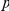 and regression coefficient (or effect size) 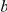. We performed simulations holding 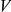 constant and sampling different allele frequencies, adjusting the effect size to obtain the desired variance explained, 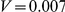. For each choice of the true model size 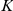 from 1 to 8, we averaged over 1000 simulations each with 8000 individuals. In each simulation, we randomly selected 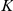 SNPs to be “causal” SNPs and distributed the variance equally across the causal SNPs, with each SNP contributing variance 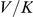. The resulting model for the phenotype 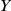 of an individual with genotype row-vector 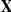 for the 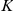 causal SNPs is 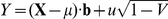, where 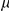 is the true population average of 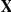, 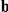 is the column-vector of SNP effects, and 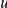 is drawn from a standard normal distribution. The resulting value for the component of 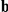 for a causal SNP with minor allele frequency 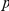 is 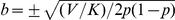. The power was calculated as (number of genes that are genome-wide significant)/1000, and the error of the estimate was calculated using 95% exact binomial confidence intervals. The p-value thresholds for genome-wide significance came from genome-wide permutations of actual data for GWiS, BimBam, minSNP-P and VEGAS. For LASSO, however, the selection index threshold from the genome-wide permutations may not be appropriate for simulations without LD. We therefore used a slightly different approach for LASSO. We calculated a null distribution of the selection index through permutations, and then used this null distribution to convert the selection index to a gene-based p-value. The p-value was then compared to the most lenient gene-based threshold of the other methods, 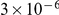 from minSNP-P.(TIF)

Figure S2 Number of SNPs and effective number of tests per gene. The number of SNPs and effective tests per gene are displayed as a density plot for (a) chromosome 1 and (b) the autosomal genome. While on average genes have 70 SNPs and 9 tests, large genes can have over 1000 SNPs and 100 tests.
(TIF)

Figure S3 Precision-recall curves for recovery of known associations. Precision and recall for recovery of 38 known associations are shown for GWiS (black), minSNP (thin blue), minSNP-P (thick blue), BIMBAM (dashed blue), LASSO (red), and VEGAS (green). Ranking is by p-value for GWiS, minSNP, minSNP-P, and VEGAS, and by Selection Index for LASSO. The tails of the curves for GWiS and LASSO are truncated when remaining loci have no SNPs entered into models, which occurs close to 50% recall. Triangles indicated the last genome-wide significant finding from each method.
(TIF)

Table S1Number of identified genome-wide significant loci. Results are reported for 20 kb and 100 kb flanking transcription boundaries. G: GWiS, S: minSNP, SP: minSNP-P, B: BIMBAM, V:VEGAS, L: LASSO. *BIMBAM was only tested for 20 kb. **VEGAS is hard-coded to use 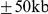.(PDF)

Table S2Genome-wide significance thresholds calculated by permutation. Results are reported for 20 kb and 100 kb flanking transcription boundaries. Thresholds for GWiS, minSNP, minSNP-P and VEGAS are for p-values. Threshold for LASSO are for the selection index. The thresholds for minSNP and LASSO decrease because the larger threshold implies more tests. GWiS and minSNP-P already include a correction for the number of tests within a gene, and thresholds are somewhat less stringent for longer gene boundaries. *BIMBAM uses the threshold from minSNP-P because both tests provide gene-based p-values with identical uniform distributions under the null. **VEGAS is hard-coded to use 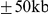.(PDF)

Table S3Top associations for PR interval. The top 100 associations are reported for GWiS, minSNP, minSNP-P, BIMBAM, VEGAS, and LASSO. The locus name concatenates the named genes within the start and end positions indicated. Additional columns provide the number of SNPs, the effective number of tests, the number of independent associations within the region (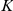), the p-value (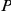), the rank from 1 through 100, and an indicator for known positives (isKnownPositive).(XLS)

Table S4 Top associations for QRS interval. The column information is the same as for Table S3.
(XLS)

Table S5 Top associations for QT interval. The column information is the same as for Table S3.
(XLS)
