# Supplementary figures and images for: Gene-Based Tests of Association
Source: PLoS Genet. 2011 Jul 28;7(7):e1002177. doi: 10.1371/journal.pgen.1002177 (PMC3145613; doi:10.1371/journal.pgen.1002177)

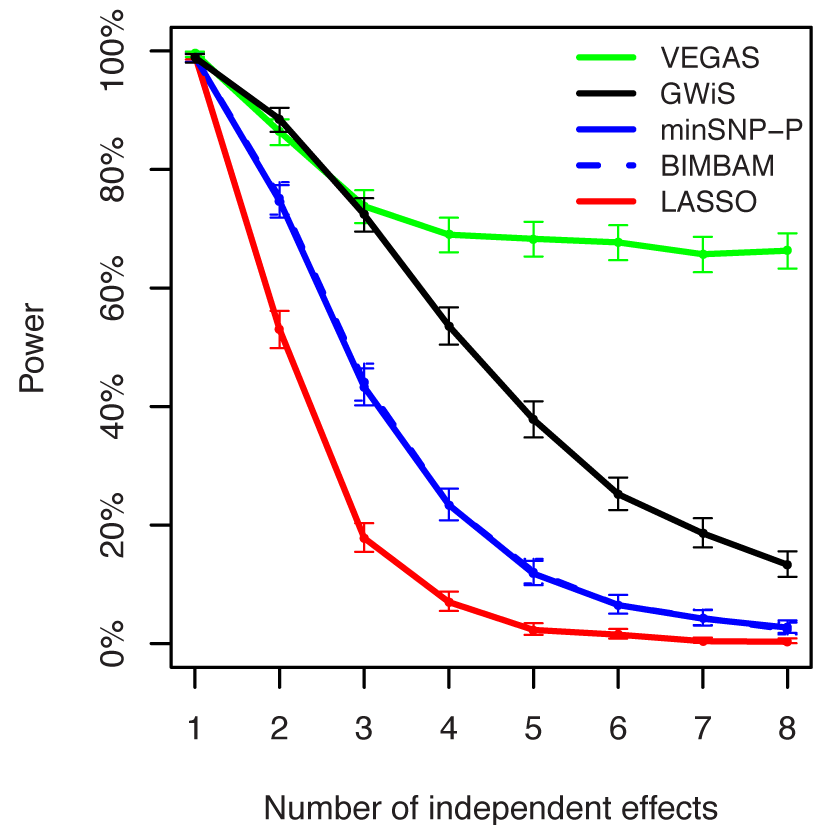

Supplement: Figure S1 — Estimated power at genome-wide significance for genotypes simulated without LD. Simulation tests were performed for true models in which a single gene housed one to eight independent causal variants. Genotypes were simulated with 20 SNPs per gene, no LD between SNPs, and minor allele frequencies selected uniformly between 0.05 and 0.5. Power estimates are provided for VEGAS (green), GWiS (black), minSNP-P (blue), BimBam (blue dashed), and LASSO (red). While VEGAS performs well in the absence of LD, its performance degrades under realistic LD (see main text, Figure 1). We simulated genetic models for quantitative traits with no linkage disequilibrium between SNPs using the simulate-qt option of PLINK. Genes were simulated with 20 SNPs and minor allele frequencies selected uniformly between 0.05 and 0.5. Genotypes were coded as allele dosages from 0 to 2. The power of a standard regression test for additive effects depends on the population variance explained, for a single variant with allele frequency and regression coefficient (or effect size) . We performed simulations holding constant and sampling different allele frequencies, adjusting the effect size to obtain the desired variance explained, . For each choice of the true model size from 1 to 8, we averaged over 1000 simulations each with 8000 individuals. In each simulation, we randomly selected SNPs to be “causal” SNPs and distributed the variance equally across the causal SNPs, with each SNP contributing variance . The resulting model for the phenotype of an individual with genotype row-vector for the causal SNPs is , where is the true population average of , is the column-vector of SNP effects, and is drawn from a standard normal distribution. The resulting value for the component of for a causal SNP with minor allele frequency is . The power was calculated as (number of genes that are genome-wide significant)/1000, and the error of the estimate was calculated using 95% exact binomial confidence intervals. Th [file pgen.1002177.s001.tif]

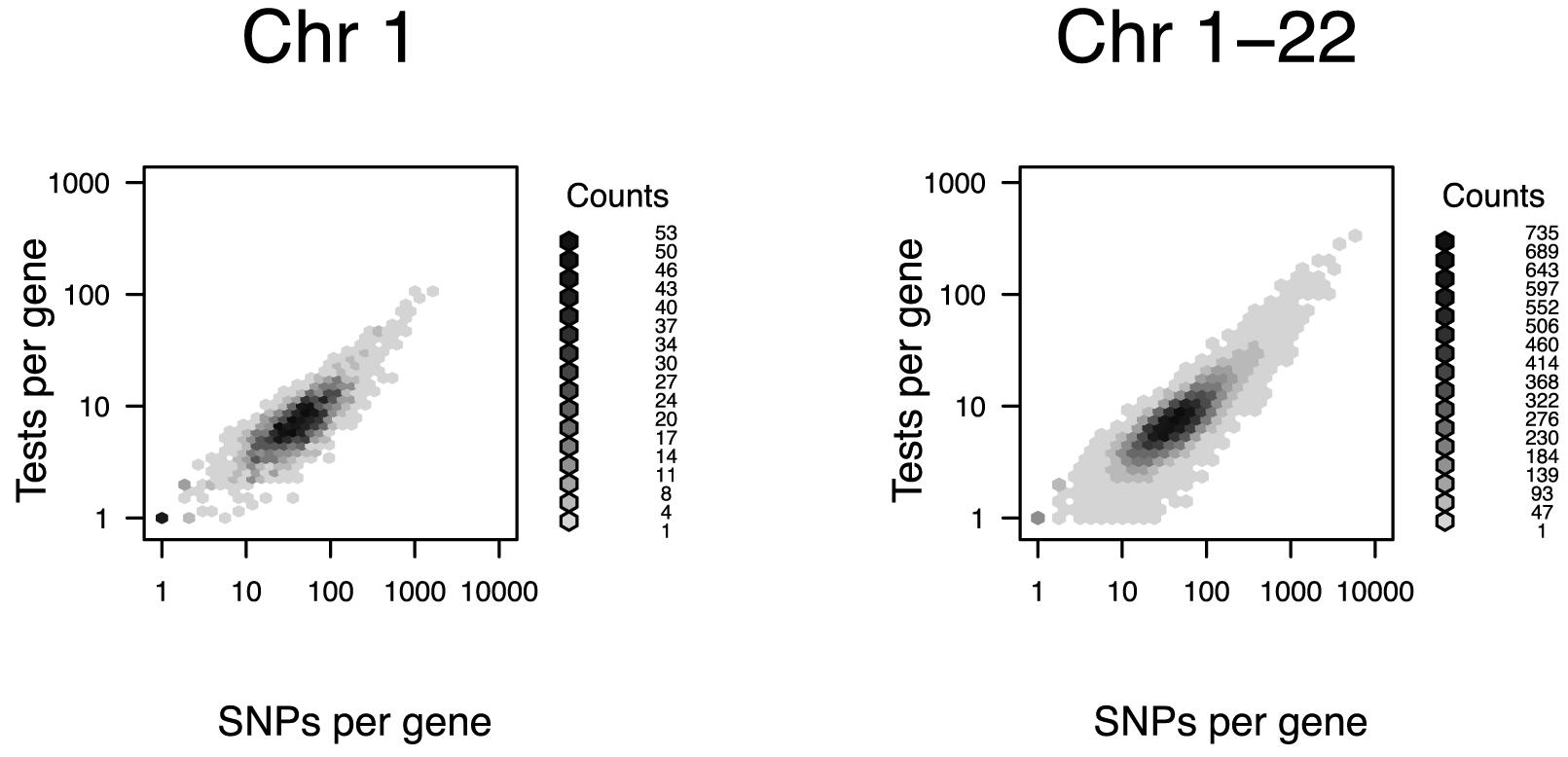

Supplement: Figure S2 — Number of SNPs and effective number of tests per gene. The number of SNPs and effective tests per gene are displayed as a density plot for (a) chromosome 1 and (b) the autosomal genome. While on average genes have 70 SNPs and 9 tests, large genes can have over 1000 SNPs and 100 tests. (TIF) [file pgen.1002177.s002.tif]

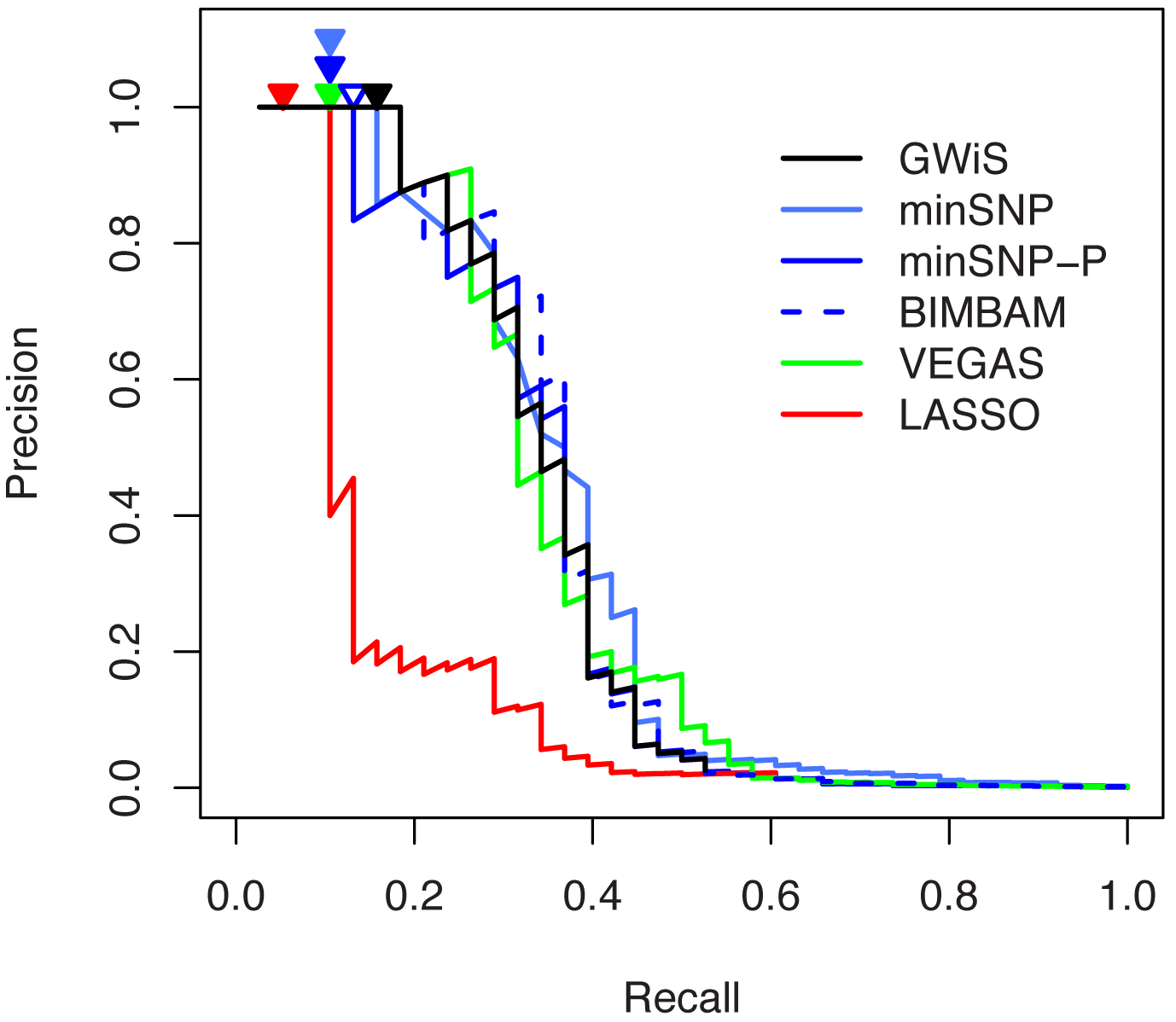

Supplement: Figure S3 — Precision-recall curves for recovery of known associations. Precision and recall for recovery of 38 known associations are shown for GWiS (black), minSNP (thin blue), minSNP-P (thick blue), BIMBAM (dashed blue), LASSO (red), and VEGAS (green). Ranking is by p-value for GWiS, minSNP, minSNP-P, and VEGAS, and by Selection Index for LASSO. The tails of the curves for GWiS and LASSO are truncated when remaining loci have no SNPs entered into models, which occurs close to 50% recall. Triangles indicated the last genome-wide significant finding from each method. (TIF) [file pgen.1002177.s003.tif]
