# Supplementary material for: Gene-Based Tests of Association
Source: PLoS Genet. 2011 Jul 28;7(7):e1002177. doi: 10.1371/journal.pgen.1002177 (PMC3145613; doi:10.1371/journal.pgen.1002177)
Supplement: Table S1 — Number of identified genome-wide significant loci. Results are reported for 20 kb and 100 kb flanking transcription boundaries. G: GWiS, S: minSNP, SP: minSNP-P, B: BIMBAM, V:VEGAS, L: LASSO. *BIMBAM was only tested for 20 kb. **VEGAS is hard-coded to use . (PDF) [file pgen.1002177.s004.pdf]

Supp. Table 1. Number of identified genome-wide significant loci.

	20 kb						100 kb			
Trait	G	S	SP	B*	V**	L	G	S	SP	L
PR	2	2	2	2	2	1	2	2	2	1
QRS	1	0	0	0	0	0	1	0	0	1
QT	3	2	2	3	2	1	3	2	2	1

Results are reported for 20 kb and 100 kb flanking transcription boundaries. G: GWiS, S: minSNP, SP: minSNP-P, B: BIMBAM, V:VEGAS, L: LASSO. *BIMBAM was only tested for 20 kb. **VEGAS is hard-coded to use ±50 kb.
