# Supplementary material for: Gene-Based Tests of Association
Source: PLoS Genet. 2011 Jul 28;7(7):e1002177. doi: 10.1371/journal.pgen.1002177 (PMC3145613; doi:10.1371/journal.pgen.1002177)
Supplement: Table S2 — Genome-wide significance thresholds calculated by permutation. Results are reported for 20 kb and 100 kb flanking transcription boundaries. Thresholds for GWiS, minSNP, minSNP-P and VEGAS are for p-values. Threshold for LASSO are for the selection index. The thresholds for minSNP and LASSO decrease because the larger threshold implies more tests. GWiS and minSNP-P already include a correction for the number of tests within a gene, and thresholds are somewhat less stringent for longer gene boundaries. *BIMBAM uses the threshold from minSNP-P because both tests provide gene-based p-values with identical uniform distributions under the null. **VEGAS is hard-coded to use . (PDF) [file pgen.1002177.s005.pdf]

Supp. Table 2. Genome-wide significance thresholds calculated by permutation.

	20 kb	100 kb
GWiS	2E-6	5E-6
minSNP	7.4E-8	4.7E-8
minSNP-P	3E-6	5E-6
BIMBAM*	3E-6	-
VEGAS**	1E-6	-
LASSO	2.1E-11	4.8E-12

Results are reported for 20 kb and 100 kb flanking transcription boundaries. Thresholds for GWiS, minSNP, minSNP-P and VEGAS are for p-values. Threshold for LASSO are for the selection index. The thresholds for minSNP and LASSO decrease because the larger threshold implies more tests. GWiS and minSNP-P already include a correction for the number of tests within a gene, and thresholds are somewhat less stringent for longer gene boundaries. *BIMBAM uses the threshold from minSNP-P because both tests provide gene-based p-values with identical uniform distributions under the null. **VEGAS is hard-coded to use ± 50 kb.
